# Forward models and passive psychotic symptoms

**DOI:** 10.3389/fnhum.2015.00022

**Published:** 2015-01-29

**Authors:** Sam Wilkinson

**Affiliations:** Philosophy Department, Durham UniversityDurham, UK

**Keywords:** forward models, auditory verbal hallucinations, psychosis, predictive processing, hypervigilance

Pickering and Clark (2014) present two ways of viewing the role of forward models in human cognition: the auxiliary forward model (AFM) account and the integral forward model (IFM) account. The AFM account “assumes a dedicated prediction mechanism implemented by additional circuitry distinct from the core mechanisms of perception and action” (p. 451). The standard AFM account exploits a corollary discharge from the motor command in order to compute the sensory consequences of the action. In contrast, on the IFM account, “perception itself involves the use of a forward (generative) model, whose role is to construct the incoming sensory signal ‘from the top down”’ (p. 453). Furthermore, within this account, motor commands are dispensed with: they are predictions that are fulfilled by movement as part of the prediction error minimization that is taken to govern all aspects of cognition (Adams et al., 2013).

Pickering and Clark present two “testing grounds” for helping us adjudicate between IFMs and AFMs, which are committed to the idea, derived from Pickering's own work, that one predicts others in a way that is similar to the way that one predicts oneself. Although I like this “prediction by simulation” account, in this commentary, I want to emphasize that neither the IFM nor the AFM accounts are necessarily wedded to it.

A less committal, and hence perhaps more compelling, testing ground is to be found in psychotic symptoms, and the capacity of the two frameworks to account for them. Indeed, using psychosis to illustrate forward modelling is not new: the inability to self-tickle was taken to be convincing data for the presence of forward models (viewed, by default, within an AFM account), and in particular for problems with them in patients with diagnoses of schizophrenia (Frith et al., 2000).

The AFM account has been used more generally to explain symptoms of schizophrenia. Something goes wrong with the generation of the forward model, and so the sensory consequences of self-generated stimuli are poorly predicted, and hence fail to be attenuated, and are, ultimately, misattributed to an external source. Although most have accepted this for delusions of control, some have questioned the application of this model to passive symptoms (Stephens and Graham, 2000), namely those which do not involve action, such as auditory verbal hallucinations (AVHs). If the symptoms of schizophrenia are explainable in terms of problems with an AFM, and this is constructed out of a motor command, then non-motoric (“passive”) symptoms cannot be so explained.

One move has been to keep working within the AFM framework but claim that “passive” symptoms merely *look* passive: they are actually “active.” Several theorists (e.g., Jones and Fernyhough, 2007) have attempted to explain AVHs in terms of inner speech misattribution, where inner speech is taken to involve motoric elements. This motoric involvement has been empirically supported by several electromyographical (EMG) studies (which measured muscular activity during inner speech) some of which date as far back as the early 1930s (Jacobsen, 1931). Later experiments made the connection between inner speech and AVH, showing that similar muscular activation is involved in healthy inner speech and AVH (Gould, 1948). The involvement of motoric elements in both inner speech and in AVH is further supported by findings (Gould, 1950) showing that when subjects hallucinated, subvocalizations occurred which could be picked up with a throat microphone. That these subvocalizations were causally responsible for the inner speech allegedly implicated in AVHs, and not just echoing it, was suggested by data (Bick and Kinsbourne, 1987) demonstrating that if people experiencing hallucinations opened their mouths wide, stopping vocalizations, then the majority of AVHs stopped.

However, this does not seem to capture all AVH subtypes. For example, Dodgson and Gordon (2009) convincingly present “hypervigilance hallucinations,” which are not based on self-generated stimuli, but constitute hypervigilant boosting and molding of external stimuli. As I have argued (Wilkinson, 2014) recently, one can account for both inner speech-based and hypervigilance hallucinations, within an IFM framework (although I called it a “Predictive Processing Framework”). Since it is good practice to support models that accommodate more phenomena, assuming (as seems plausible) that hypervigilance hallucinations are a genuine subtype of AVH, the IFM account is preferable to the AFM account.

In conclusion, although I agree with Pickering and Clark that the IFM account is preferable, I do so on the basis of a somewhat different “testing ground.” The IFM can account for both active and passive psychotic symptoms. The AFM, in tying itself to motor commands, can only explain the former.

## Conflict of interest statement

The author declares that the research was conducted in the absence of any commercial or financial relationships that could be construed as a potential conflict of interest.
